# Risk of *Plasmodium falciparum* infection in south-west Burkina Faso: potential impact of expanding eligibility for seasonal malaria chemoprevention

**DOI:** 10.1038/s41598-022-05056-7

**Published:** 2022-01-26

**Authors:** Jean Baptiste Yaro, Alfred B. Tiono, Alphonse Ouedraogo, Ben Lambert, Z. Amidou Ouedraogo, Amidou Diarra, Adama Traore, Malik Lankouande, Issiaka Soulama, Antoine Sanou, Eve Worrall, Efundem Agboraw, N’Fale Sagnon, Hilary Ranson, Thomas S. Churcher, Steve W. Lindsay, Anne L. Wilson

**Affiliations:** 1 Centre National de Recherche et de Formation sur le Paludisme, Ouagadougou, Burkina Faso; 2 Department of Biosciences, Durham University, Durham, UK; 3 MRC Centre for Global Infectious Disease Analysis, School of Public Health, Faculty of Medicine, Imperial College London, London, UK; 4 Institute of Biodiversity, Animal Health & Comparative Medicine, Glasgow University, Glasgow, UK; 5 Department of Vector Biology, Liverpool School of Tropical Medicine, Liverpool, UK; 6 Institut de Recherche en Sciences de la Santé, Ouagadougou, Burkina Faso

**Keywords:** Risk factors, Epidemiology, Malaria

## Abstract

Burkina Faso has one of the highest malaria burdens in sub-Saharan Africa despite the mass deployment of insecticide-treated nets (ITNs) and use of seasonal malaria chemoprevention (SMC) in children aged up to 5 years. Identification of risk factors for *Plasmodium falciparum* infection in rural Burkina Faso could help to identify and target malaria control measures. A cross-sectional survey of 1,199 children and adults was conducted during the peak malaria transmission season in the Cascades Region of south-west Burkina Faso in 2017. Logistic regression was used to identify risk factors for microscopically confirmed *P. falciparum* infection. A malaria transmission dynamic model was used to determine the impact on malaria cases averted of administering SMC to children aged 5–15 year old. *P. falciparum* prevalence was 32.8% in the study population. Children aged 5 to < 10 years old were at 3.74 times the odds (95% CI = 2.68–5.22, *P* < 0.001) and children aged 10 to 15 years old at 3.14 times the odds (95% CI = 1.20–8.21, *P* = 0.02) of *P. falciparum* infection compared to children aged less than 5 years old. Administration of SMC to children aged up to 10 years is predicted to avert an additional 57 malaria cases per 1000 population per year (9.4% reduction) and administration to children aged up to 15 years would avert an additional 89 malaria cases per 1000 population per year (14.6% reduction) in the Cascades Region, assuming current coverage of pyrethroid-piperonyl butoxide ITNs. Malaria infections were high in all age strata, although highest in children aged 5 to 15 years, despite roll out of core malaria control interventions. Given the burden of infection in school-age children, extension of the eligibility criteria for SMC could help reduce the burden of malaria in Burkina Faso and other countries in the region.

## Introduction

Impressive reductions in malaria occurred throughout sub-Saharan Africa from 2000 to 2015^1^. Progress, however, has not been geographically uniform. In many high-burden countries, parasite prevalence rates and mortality from malaria remain obstinately high despite roll out of key malaria control interventions including insecticide-treated nets (ITNs), use of seasonal malaria chemoprevention (SMC) in children aged less than five years, and diagnosis and treatment using artemisinin combination therapy (ACTs)^2^.

Burkina Faso has highly intense and seasonal malaria transmission and is one of the ten countries in the world with the highest burden of malaria^3^. It has been designated as a High Burden High Impact country by the World Health Organisation (WHO) with calls for a more aggressive approach to malaria control^3^. Burkina Faso has seen increases in malaria cases from 7.8 million cases in 2014^4^ to 11.5 million cases in 2018 and 11.3 million cases in 2020, according to the National Malaria Control Programme (NMCP)^5,6^. Malaria remains the main cause of outpatient attendance at health centres in all age groups, and severe malaria was responsible for 69% of deaths in children aged 1 to 15 years in medical centres and hospitals in 2020^6^. Increases in malaria cases are being observed despite national ITN distribution campaigns in 2010, 2013, 2016 and 2019 and introduction of seasonal malaria chemoprevention (SMC) in children aged 3 to 59 months since 2014. SMC (previously termed intermittent preventive treatment in children) requires the administration of the anti-malarial drugs sulfadoxine-pyrimethamine (SP) and amodiaquine (AQ) monthly for four months during the high transmission season to prevent malaria and is recommended by WHO across the Sahel, where transmission is highly seasonal^7^. Potential explanations for the stagnating progress in malaria control include low ITN usage or lack of ITN bioefficacy and durability^8–11^, a rise in the resistance of vectors to pyrethroid insecticides used to treat ITNs^12–14^, and an increase in outdoor vector biting^14^.

A cross-sectional survey was carried out in 2017 to determine the prevalence of *P. falciparum* infection in different age groups and identify risk factors in south-west Burkina Faso. These data were used to parameterise a mathematical model of malaria transmission to explore how expanding the age range for using SMC would impact the malaria burden in the study area.

## Methods

### Study design

A cross-sectional survey of *P. falciparum* prevalence and risk factors for infection was conducted in October and November 2017 shortly after the peak of malaria transmission.

### Study site

**Figure 1:**
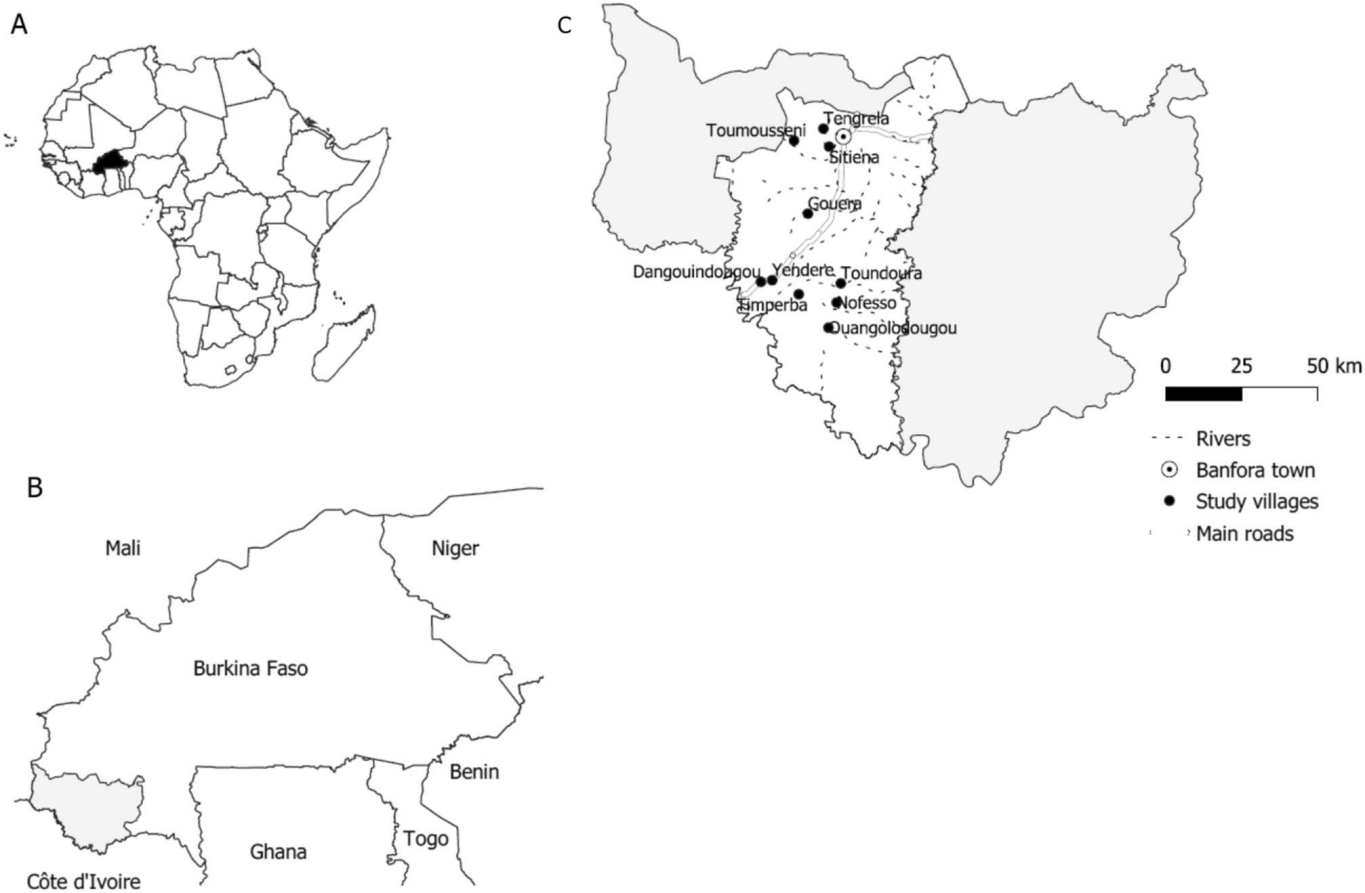
Map of the 10 study villages: (**A**) location of Burkina Faso, (**B**) location of Cascades Region in Burkina Faso and (**C**) location of Banfora Health District and study villages in Cascades Region. The map was generated using QGIS 3.16^19^. Background layers were downloaded for OpenStreetMap^20^, villages were digitised by the authors using GPS coordinates collected in the field using a GARMIN eTREX 10 GPS.

The study was conducted in ten villages in the Banfora Health District, an area of Sudanian savannah in the Cascades region, south-west Burkina Faso (lying between 10°40’ to 10°04′13’’ north latitude and 5°01′21’’ to 4°46′18’’ west longitude) with a population of 392,498 in 2017 (Fig. 1) . The study site experiences intense seasonal malaria transmission with peaks during the rainy season, from May to November^15^, with most cases occurring in September^16^. *Plasmodium falciparum* accounts for 90% of cases^15^ and the main malaria vectors are *Anopheles gambiae*, *An. coluzzii* and, to a lesser extent, *An. arabiensis*^14,17^. The site has an entomological inoculation rate (EIR) of 80 infective bites per child during the transmission season^8^, with another study suggesting up to 52 infective bites per person per night during the peak transmission season in October^18^. The NMCP undertook an ITN universal coverage campaign (distribution of one ITN for two persons) in 2010, 2013 and 2016 using nets either treated with permethrin or deltamethrin. No additional nets were distributed by the study team. Indoor residual spraying was not conducted during the study or the preceding 12 months. In Burkina Faso since 2014, children aged 3 to 59 months receive SMC on four occasions during the transmission season using SP-AQ as per WHO recommendations^7^.

### Sampling design

A random sample of 10 villages were selected from a list of villages in the study area using a two-stage process. Five health centres in the study area were chosen with each health centre having a catchment radius of approximately 10 km. Two villages were randomly selected from each catchment area, giving a total of 10 villages, at least 3 km apart. Permission to enter the communities was sought from village leaders.

An age-stratified cross-sectional survey of both children and adults in three age groups (2 to < 10 years, 10 to < 30 years, ≥ 30 years) was conducted to determine the prevalence of *P. falciparum*. The survey aimed to sample 1,200 individuals, 400 from each of the three age strata. 150 study subjects (50 in each age strata) were randomly selected from the Health and Demographic Surveillance System (HDSS) lists of the 10 study villages and entered the screening process. Each study subject was selected from a different household. The first 40 individuals per age strata and per village who provided informed consent were enrolled. Participants were excluded if they were currently participating in a trial of a malaria vaccine or drug, under chemoprophylaxis (except for SMC) or currently participating in a related cohort study^8^.

### *P. falciparum* infection survey

A finger-prick blood sample was taken from each participant. Two blood slides were prepared and a malaria rapid diagnostic test (RDT, SD BIOLINE Malaria Ag *P.f*/Pan screening test Abbott, Geonggi-do, Republic of Korea) performed for point-of-care diagnosis of those with fever (axillary temperature ≥ 37.5 °C) or history of fever in the past 48 h. Individuals with positive RDTs were offered treatment with artemether-lumefantrine (AL) according to national guidelines^21^. Thick blood films were stained with Giemsa and examined under 100-fold magnification by experienced microscopists centrally at Centre National de Recherche et de Formation sur le Paludisme (CNRFP) in Banfora. Parasite counts were recorded per high power field and 100 fields counted before a slide was declared negative. Each slide was read separately by two independent microscopists. Discrepancies in positive and negative reads and parasite counts differing by more than tenfold between the two reads were resolved by a third reader. The final result was average of the two-closer readings. Blood spots were also taken for polymerase chain reaction analysis but unfortunately due to a lack of amplification we did not obtain results.

### Risk factor survey

At the same time as the infection survey, a questionnaire was administered to the study participant (or caregiver for children, as indicated) to gather information on demographics (age, gender, ethnicity, religion, education, occupation), use of malaria control measures (bednet use the previous night, use of topical or household insecticides e.g. insecticide aerosols, mosquito coils in the past week, receipt of SMC in previous month), house construction (roof material, whether the space between the wall and the roof, i.e. the eaves, were closed), presence of electricity or functioning fan, presence of animals within 5 m of the household, and travel history outside of the village in the previous two weeks. Information was also collected from the study participant or the head of household (as indicated) on asset ownership and household characteristics, following standard procedures used in the Burkina Faso Demographic and Health Survey (DHS)^22^.

Human landing catches were carried out inside houses during the 2017 transmission season (1st June to 17th December) in each of the 10 villages and are a subset of results previously published by Sanou *et al*^14^. Four randomly selected households were sampled on one night every month (total of 187 collections due to public holidays), with a different group of households selected the following month to maximise spatial coverage. Households selected for entomological sampling were not necessarily the households of participants in the cross- sectional survey. Volunteers were recruited from the villages and trained to collect mosquitoes landing on their legs between 19.00 h to 06.00 h. Mosquitoes were typed to species using established morphological keys. Phenotypic insecticide resistance was measured using WHO tube tests as per standard procedures^23^. Assays were performed with *An. gambiae* s.l. mosquitoes reared from larvae collected in seven study villages during the 2017 transmission season (because of limited availability of larval habitats in the other three villages during the period of the survey)^14^.

### Sample size considerations

A random sample of 400 individuals from each of the three age groups (2 to < 10 years, 10 to < 30 years, ≥ 30 years) were selected from 10 villages giving a total sample size of 1,200 individuals. Assuming a true parasite prevalence ranging between 40 to 60% across the three age groups^24^, the study was able to measure the point prevalence of *P. falciparum* infection by microscopy with a 5% precision at the 95% confidence level^25^. No sample size calculation was performed for the mosquito collections and instead the number of households and trapping nights were determined based on logistical constraints.

### Data management and statistical analysis

Data were collected on personal digital assistants (PDAs) programmed with an electronic data capture system, Kobo Collect (Version 1.4.8). Forms were piloted in the field prior to use and had drop-down boxes and consistency checks to avoid data entry errors. PDAs were uploaded by fieldworkers weekly to a central computer.

The primary outcome was *P. falciparum* infection confirmed by microscopy (any level of parasite density). Secondary outcomes were: (i) prevalence of symptomatic malaria defined as axillary temperature ≥ 37.5 °C (or history of fever within the previous 48 h) with microscopically confirmed *P. falciparum* infection and (ii) prevalence of high-density *P. falciparum* infection (> 5,000 parasites/µL) detected by microscopy.

Principal component analysis was used to calculate the socio-economic status (SES) factor score (based on asset ownership and household characteristics). SES factor scores were ranked and households divided into five equal wealth quintiles (1 being the poorest, through to 5, least poor). The EIR or estimated number of infectious bites per study participant during the transmission season was calculated using the formula *EIR* = *Ma* × *S* × *d* where *Ma* is the human biting rate, estimated from the arithmetic mean number of female *An. gambiae* s.l. caught per human landing catches across the six-month transmission season, where *S* is the proportion of female *An. gambiae* s.l. found to be sporozoite positive by village and *d* is the number of days in the transmission season.

Mean values were compared using a t-test and proportions compared using chi-squared tests. Parasite prevalence was estimated as the proportion of subjects infected divided by the number of subjects tested. Logistic regression was used to investigate the association between malaria infection and risk factors, adjusting for clustering by village. Univariate analysis was conducted followed by construction of a simple multivariate model in which every risk factor was included, irrespective of whether the variable was significant in the univariate model. All analyses were carried out using Stata 15 (Statacorp, Texas, USA).

### Malaria transmission dynamic model

A widely used transmission dynamics mathematical model of malaria was used to investigate the impact of modifying SMC in the Cascades region of Burkina Faso. The individual-based stochastic model mechanistically captures transmission of *P. falciparum* malaria in humans and *Anopheles* mosquito vectors. All differential equations describing the dynamics of the infection in populations with malaria control interventions have been comprehensively reported in Griffin et al.^26^ and Winskill et al.^27^ whilst the model code is available from https://github.com/jamiegriffin/Malaria_simulation. This model captures the age distribution of infection in areas with different levels of endemicity^28^ and has been used to investigate the impact of SMC^29^. Here we use a version of the model calibrated for the region (Lambert et al., unpublished) which is parameterised with local epidemiological^8^ and entomological data^14,18^ and fitted to estimates of malaria prevalence (assessed by microscopy) across the Cascades region collated by the Malaria Atlas Project (https://malariaatlas.org/). The calibration process uses the method of maximum likelihood (assuming a beta-binomial model) to select a value of the mosquito-to-human density parameter (and corresponding over-dispersion parameter of the beta-binomial). It is used to predict the number of clinical cases per person in the whole population between 2019 and 2022 following the mass ITN campaign in 2019. Results are averaged over the three-year period (the time between ITN campaigns) as cases will depend on ITN use which drops following mass distribution. The type of nets distributed varied at the district level within the Cascades region. Here we assume that the population received either pyrethroid-only or pyrethroid-piperonyl butoxide (PBO) ITNs^30^. PBO is an insecticide synergist which inhibits the action of resistance-associated metabolic enzymes of the cytochrome P450 family and improves control of pyrethroid-resistant anopheline mosquitoes. The added advantage of pyrethroid-PBO ITNs over pyrethroid-only ITNs is estimated from a meta-analyses of experimental hut trial data^31^ and the level of resistance for the region estimated using discriminating dose bioassays^14^. The impact of changing SMC is assessed using the same method as outlined previously^32^, either halting SMC, maintaining the existing age range of 3–59 months or extending the upper age limit to 10 or 15 years. In all simulations with SMC, coverage is assumed to match previous years (81% of children receiving treatment each round)^2^.

### Ethical consideration

Adult study participants and the caregivers of children aged under 20 years provided informed consent to participate in the cross-sectional survey. In addition, minors aged 12 to 19 years were required to provide written assent before they could be enrolled in the study. All the consenting and assenting processes were conducted in the presence of an impartial witness if the participant was illiterate. Ethical approval for this study was provided by the Burkina Faso Health Research Ethics Committee (Deliberation No 2016–12-137), CNRFP Institutional Bioethics Committee (No2016/000007/MS/SG/CNRFP/CIB), Durham University’s Department of Biosciences Ethics Committee (SBBS/EC/MIRA) and Liverpool School of Tropical Medicine Ethical Committee (Protocol number: 16/047). The study was conducted in compliance with principles set out by the International Conference on Harmonization Good Clinical Practice, the Declaration of Helsinki and the regulatory requirements of Burkina Faso.

## Results

### Socio-demographic characteristics of the survey participants

A total of 1,199 individuals were surveyed, 399 (33.3%) were aged 2 to < 10 years, 397 (33.1%) aged 10 to < 30 years and 403 (33.6%) aged ≥ 30 years (Table 1). 55.0% of the study population were female (660/1199). The most common ethnic groups were Gouin and Turka (51.9%, 622/1199) and Karaboro (18.8%, 226/1199). The respondents were Muslim (35.4%, 784/1199), Animists (20.3%, 243/1199) and Christians (14.3%, 172/1199). Most study participants were illiterate (70.5%, 845/1199) and farmers (54.8%, 657/1199), whereas 43.5% (522/1199) of participants were not engaged in income generating work either because they were too young or because they were too old to work. 90.7% of study participants (1,087/1,199) reported sleeping under a bednet the previous night. 9.2% (110/1199) of the population reported using a topical repellent in the last week, while 6.2% (74/1199) reported using household insecticides (e.g. insecticide aerosols, mosquito coils). Only 1.8% (22/1199) of the study population reported travelling outside their village in the two weeks before the survey. The EIR in the study area was 188 bites per person over the transmission season ranging from 0 in Sitiena village to 336 in Dangouindougou village.

**Table 1:** Characteristics of participants of the cross-sectional survey.

Characteristic	Overall, N (%) N = 1199	2 to < 10 years, n = 399	10 to < 30 years, n = 397	> 30 years, n = 403
Age (mean / SD)	23.7 (19.1)	5.9 (2.3)	18.0 (5.7)	46.9 (12.8)
Female gender	660 (55.0)	191 (47.9)	214 (53.9)	255 (63.3)
**Ethnicity**				
Gouin/Turka	622 (51.9)	213 (53.4)	197 (49.6)	212 (52.6)
Karaboro	226 (18.8)	73 (18.3)	72 (18.1)	81 (20.1)
Other ethnic group	351 (29.3)	113 (28.3)	128 (32.2)	110 (27.3)
**Religion**				
Muslim	784 (65.4)	255 (63.9)	270 (68.0)	259 (64.3)
Christian	172 (14.3)	60 (15.0)	58 (14.6)	54 13.4)
Animist	243 (20.3)	84 (21.1)	69 (17.4)	90 (22.3)
**Education**				
Illiterate	845 (70.5)	298 (74.7)	201 (50.6)	346 (86.1)
Literate	353 (29.5)	101 (25.3)	196 (49.4)	56 (13.9)
**Occupation**				
Farming	657 (54.8)	40 (10.0)	245 (61.7)	372 (92.3)
Commercial and office workers	20 (1.7)	1 (0.3)	6 (1.5)	13 (3.2)
None or retired	522 (43.5)	358 (89.7)	146 (36.8)	18 (4.5)
Used any bednet the previous night	1087 (90.7)	376 (94.2)	346 (87.2)	365 (90.6)
History of travel in the previous 2 weeks	22 (1.8)	9 (2.3)	5 (1.3)	8 (2.0)
Received SMC in previous month	169 (42.4)			
Eave construction of the sleeping room of the subject*				
Closed	789 (65.8)	276 (69.2)	273 (68.8)	240 (59.6)
Open	198 (16.5)	41 (10.3)	74 (18.6)	83 (20.6)
Roof construction of the sleeping room of the subject*				
Metal	689 (57.5)	217 (54.4)	229 (57.7)	243 (60.3)
Non-metal (Thatch)	298 (24.9)	100 (25.1)	118 (29.7)	80 (19.9)
Sleeping room of subject has a functioning lightbulb installed*	763 (63.6)	274 (68.7)	247 (62.2)	242 (60.0)
Sleeping room of subject has a functioning fan	74 (6.2)	34 (8.5)	16 (4.0)	24 (6.0)
Animals tethered within 5 m of this part of the house*	65 (5.4)	27 (6.8)	19 (4.8)	19 (4.7)
Use of topical repellent in last week	110 (9.2)	38 (9.5)	32 (8.1)	40 (9.9)
Use of household insecticides in the last week*	74 (6.2)	22 (5.5)	24 (6.0)	28 (6.9)

*Missing data.

### Malariometric characteristics of the survey participants

**Figure 2:**
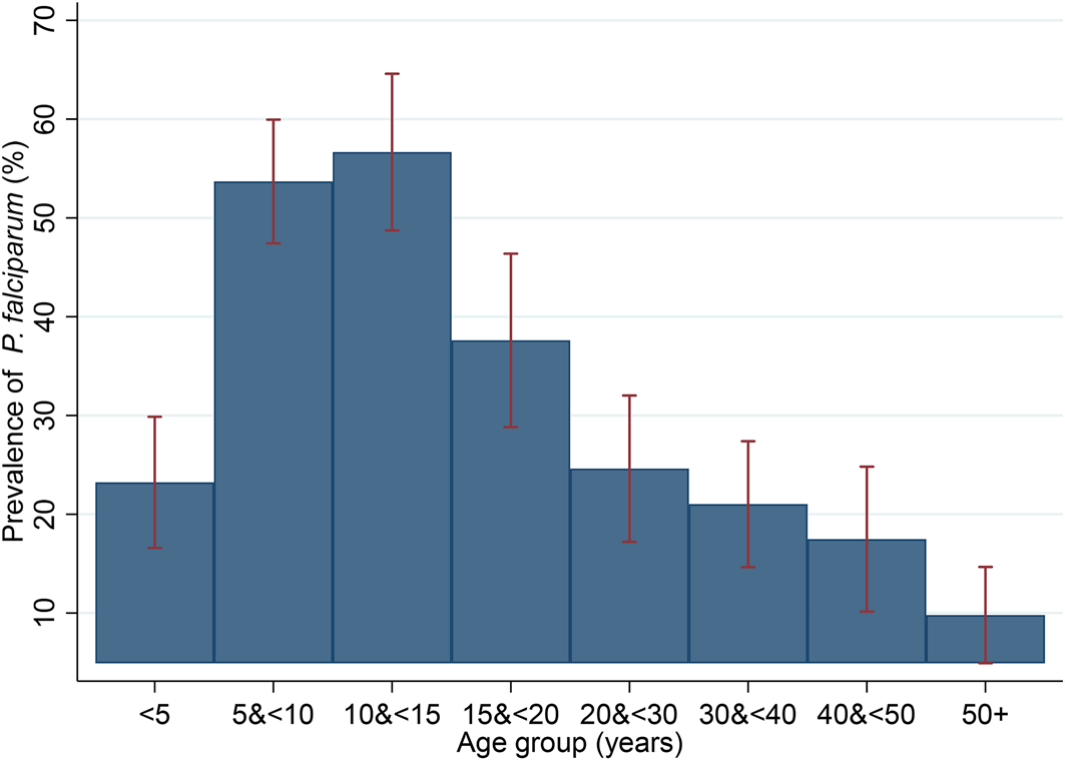
Prevalence of *P. falciparum* infection by age group in study population. Error bars are 95% confidence intervals.

Overall *P. falciparum* prevalence detected by microscopy was 32.8% (393/1199) in the study area (Table 2). Prevalence of *P. falciparum* was highest in children aged 5 to < 10 years old (53.7%) and 10 to < 15 years old (56.7%) compared to other age strata, with these two age groups responsible for 55.0% of the parasite burden in the population (216/393 *P. falciparum* positive participants) (Fig. 2). Overall, 3.3% (40/1199) of individuals had high density *P. falciparum* infections (> 5,000 parasites/µL), with prevalence highest in children aged 2 to < 5 years old (9.0%, 14/155) and 5 to < 10 years old (9.0%, 22/244) (Table 2). None of those aged > 30 years had high density *P. falciparum* infections. Geometric mean density of P. falciparum parasites was highest in children aged 2 to < 5 years at 3333.9 (1924.2–5776.3). Overall, the *P. falciparum* gametocyte prevalence was 4.8% (57/1199) with slightly higher prevalence among children < 15 years than in older age groups. Only a few individuals harboured other *Plasmodium* species with 0.8% (9/1199) infected with *P. malariae* and 1.7% (20/1199) with mixed *P. falciparum* and *P. malariae* infection.

**Table 2:** Parasitological characteristics of cross-sectional survey participants.

Variable		Overall N = 1199	2 to < 5 years N = 155	5 to < 10 years N = 244	10 to < 15 years N = 150	15 to < 20 years N = 117	> 20 years N = 533
*Plasmodium* infection							
*P. falciparum*	n (%)	393 (32.8)	36 (23.2)	131 (53.7)	85 (56.7)	44 (37.6)	97 (18.2)
*P. malariae*	n (%)	9 (0.8)	0	7 (2.9)	1 (0.7)	0	1 (0.2)
*Mixed infection (P. falciparum* + *P. malariae)*	n (%)	20 (1.7)	2 (1.3)	12 (4.9)	4 (2.7)	1 (0.9)	1 (0.2)
*P. ovale*	n (%)	0	0	0	0	0	0
*P. vivax*	n(%)	0	0	0	0	0	0
*P. falciparum* high density (> 5,000 parasites/µL) prevalence, detected by microscopy	n (%)	40 (3.3)*	14 (9.0)	22 (9.0)	4 (2.7)	0	0
Geometric mean of *P. falciparum* density per μL	(95%CI)	573.5 (491.0–669.7)	3333.9 (1924.2–5776.3)	882.0 (682.7–1139.5)	507.9 (385.9–668.4)	294.9 (200.6–433.6)	234.3 (182.6–300.6)
Gametocyte prevalence *P.f* (any density)	n (%)	57 (4.8)	12 (7.7)	17 (7.0)	10 (6.7)	3 (2.6)	15 (2.8)
Geometric mean *P.f* gametocytes density per μL	(95% CI)	31.3 (22.8–43.0)	66.1 (25.1–174.3)	40.2 (21.1–76.6)	20.6 (10.1–41.9)	35.4 (11.6–108.4)	16.7 (12.0–23.3)
Fever (body temperature ≥ 37.5 °C or history of fever in the previous 48 h)	n (%)	80 (6.7)	8 (5.2)	14 (5.7)	13 (8.7)	5 (4.3)	40 (7.5)
Prevalence of symptomatic *P. falciparum* malaria (fever + microscopy positive)	n (%)	32 (2.7)	3 (1.9)	9 (3.7)	9 (6.0)	3 (2.6)	8 (1.5)
Prevalence of asymptomatic *P. falciparum* infection	n (%)	361 (30.1)	33 (21.3)	122 (50.0)	76 (50.7)	41 (35.0)	89 (16.7)

*2/40 were mixed *P.f*/*P.m* infections.

Prevalence of symptomatic *P. falciparum* malaria (those with fever or reported fever within 48 h with microscopically confirmed parasitaemia) was 2.7% (32/1199) in the study population. Asymptomatic *P. falciparum* infection was found in 30.1% (361/1199) of all study participants, and was highest in children aged 5 to < 10 years (50.0%, 122/244) and 10 to < 15 years old (50.7% 76/150).

### Risk factors for *P. falciparum* infection in survey participants

In the multivariate analysis, children aged 5 to < 10 years old were at 3.74 times the odds (95% CI = 2.68–5.22, *P* < 0.001) and those aged 10 to 15 years old at 3.14 times the odds (95% CI = 1.20–8.21, *P* = 0.02) of *P. falciparum* infection compared to children aged less than 5 years old (Table 3). Reporting sleeping under an ITN the previous night was associated with 0.47 times the odds of *P. falciparum* infection (95% CI = 0.31–0.71, *P* < 0.001), compared to those that did not report using an ITN. There was evidence of reduced odds of *P. falciparum* infection among individuals that reported travel in the previous fortnight (OR = 0.51, 95% CI = 0.30–0.87, *P* = 0.01) and individuals sleeping in a room with electric fan (OR = 0.39, 95% CI = 0.18–0.81, *P* = 0.01), although numbers of those reporting travel in the past 2 weeks and sleeping with a fan were relatively few (4/1199 and 74/1199, respectively). No association was found between risk of *P. falciparum* infection and gender, ethnicity, education, occupation, religion, socio-economic status, eave status, roof material, presence of an electric light in the sleeping room, use of topical repellent or household insecticides, having animals tethered within 5 m of the house, EIR and % mortality in a WHO tube test against 0.05% deltamethrin.

**Table 3:** Risk factors for *P. falciparum* infection in study participants.

Risk factors	*P. falciparum* prevalence n/N (%)	Univariate analysis^a^		Multivariate analysis^b^	
		Crude OR (95%CI)	*P*-value	Adjusted OR (95%CI)	*P*-value
**Age (years)**					
2 to < 5	36/155 (23.2)	1	-	1	
5 to < 10	131/244 (53.7)	3.83 (2.48**–**5.91)	< 0.001	3.74 (2.68**–**5.22)	< 0.001
10 to < 15	85/150 (56.7)	4.32 (2.19**–**8.53)	< 0.001	3.14 (1.20**–**8.21)	0.02
15 to < 20	44/117 (37.6)	1.99 (1.17**–**3.40)	0.01	1.31 (0.81**–**2.12)	0.28
≥ 20	97/533 (18.2)	0.74 (0.51**–**1.07)	0.11	0.45 (0.26**–**0.75)	0.002
**Gender**					
Male	199/539 (36.9)	1		1	
Female	194/660 (29.4)	0.71 (0.58**–**0.87)	0.001	0.79 (0.56**–**1.10)	0.16
**Ethnicity**					
Gouin and Turka	214/622 (34.4)	1		1	
Karaboro	65/226 (28.8)	0.77 (0.57**–**1.04)	0.09	1.02 (0.69**–**1.51)	0.91
Other ethnic groups	114/351 (32.5)	0.92 (0.63**–**1.34)	0.66	0.95 (0.57**–**1.57)	0.85
**Education**					
Illiterate	232/845 (27.5)	1		1	
Literate	161/353 (45.6)	2.22 (1.92**–**2.56)	< 0.001	1.15 (0.93**–**1.44)	0.20
**Occupation**					
None / retired	217/522 (41.6)	1		1	
Farmer / pastoral sector	171/657 (26.0)	0.49 (0.38**–**0.65)	< 0.001	1.42 (0.94**–**2.17)	0.10
Commercial and Office worker	5/20 (25.0)	0.47 (0.18**–**1.25)	0.13	1.16 (0.30**–**4.52)	0.83
**Religion**					
Muslim	253/784 (32.3)	1		1	
Christian	56/172 (32.6)	1.01 (0.61**–**1.67)	0.96	0.78 (0.50**–**1.22)	0.37
Animist	84/243 (34.6)	1.11 (0.86**–**1.44)	0.43	0.88 (0.64**–**1.21)	0.44
**Socio-Economic Status quintile**					
Quintile 1 (lowest)	79/237 (33.3)	1		1	
Quintile 2 (low)	81/238 (34.0)	0.96 (0.91**–**1.02)	0.21	0.99 (0.89**–**1.09)	0.82
Quintile 3 (middle)	84/239 (35.1)				
Quintile 4 (high)	72/235 (30.6)				
Quintile 5 (highest)	73/237 (30.8)				
**History of travel in the previous 2 weeks**					
No	389/1177 (33.1)	1		1	
Yes	4/22 (18.2)	0.45 (0.22**–**0.94)	0.03	0.51 (0.30**–**0.87)	0.01
**Slept under a bednet the previous night**					
No	44/111 (39.6)	1		1	
Yes	348/1087 (32.0)	0.72 (0.47**–**1.10)	0.13	0.47 (0.31**–**0.71)	< 0.001
**Eave construction of the sleeping room of the subject**					
Closed	258/789 (32.7)	1		1	
Open	59/198 (29.8)	0.87 (0.52**–**1.45)	0.60	1.55 (0.99**–**2.41)	0.05
**Roof construction of the sleeping room of the subject**					
Metal	229/689 (33.2)	1		1	
Thatch/mud	88/298 (29.5)	0.84 (0.62**–**1.13)	0.26	1.10 (0.61**–**1.96)	0.76
**Sleeping room of subject has a functioning lightbulb installed**					
No	67/223 (30.0)	1		1	
Yes	250/763 (32.8)	1.13 (0.89**–**1.45)	0.31	1.23 (0.78**–**1.93)	0.37
**Sleeping room of subject has a functioning fan**					
No	301/913 (33.0)	1		1	
Yes	16/74 (21.6)	0.56 (0.38**–**0.82)	0.003	0.39 (0.18**–**0.81)	0.01
**Animals tethered within 5 m of the house**					
No	298/922 (32.3)	1		1	
Yes	19/65 (29.2)	0.86 (0.44**–**1.68)	0.67	1.12 (0.54**–**2.33)	0.76
**Use of topical repellent in last week**					
None	355/1089 (32.6)	1		1	
Yes	38/110 (34.5)	1.09 (0.77**–**1.55)	0.62	0.95 (0.42**–**2.12)	0.90
**Use of household insecticides in the last week**					
None	290/913 (31.8)	1		1	
Yes	27/74 (36.5)	1.23 (0.99**–**1.54)	0.07	1.68 (0.79**–**3.61)	0.18
EIR (village-level)		1.00 (1.00**–**1.00)	0.03	1.00 (0.99**–**1.00)	0.76
% mortality in WHO tube test against 0.05% deltamethrin		1.65 (1.21**–**2.25)	0.002	2.10 (0.82**–**5.37)	0.12

^a^Adjusted for clustering by village.

^b^Adjusted for clustering by village and all other factors in model.

### Potential impact of extending the age eligibility for SMC

**Figure 3:**
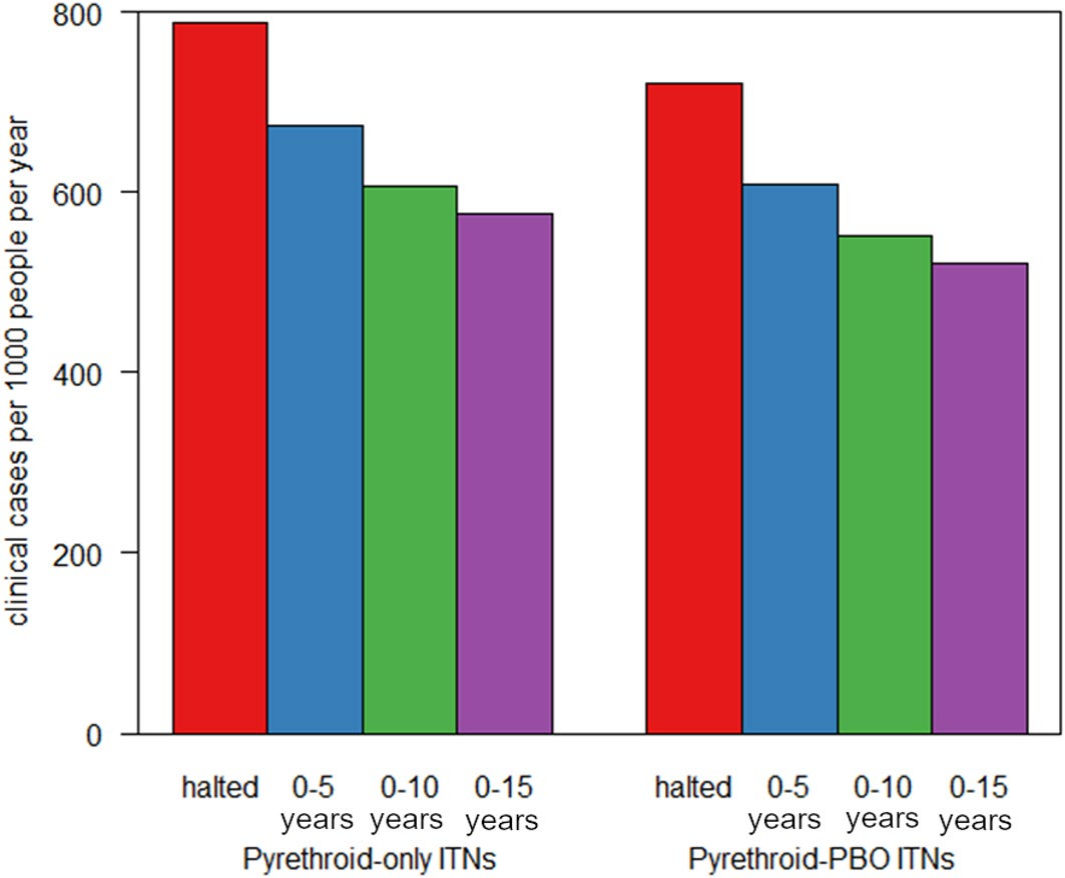
Clinical incidence of malaria predicted by either halting or extending the age range of the annual SMC campaign. Model predictions for the average number of clinical cases per year per 1000 people if SMC was halted (red bar), continued implementation across the existing target age (0–5 years, blue bar) or extended to 0–10 years old (green bar) or 0–15 years (purple bar). Incidence is averaged over the whole population (all ages) over a three-year period following the change in policy to reflect the regularity of mass ITN campaigns. ITNs are assumed to be either pyrethroid-only ITNs (left bars) or pyrethroid- piperonyl butoxide (PBO) ITNs (right bars).

The transmission modelling indicates that at 91% usage of pyrethroid ITNs, administration of SMC to children aged under 5 years reduces all-age clinical malaria case incidence by 14.7% from 789 cases per 1000 people/year to 673 cases/1000 people/year in the Cascades Region (assuming 81% coverage of SMC) (Fig. 3). Extending the age eligibility to children under 10 years old would reduce clinical malaria case incidence by a further 10% (to 606 cases/1000 people/year). Extending SMC eligibility to children under 15 years old would reduce case incidence by 14.6% to 575 cases/1000 people/year. Assuming coverage of pyrethroid-PBO nets, administration of SMC to children under 10 years old would reduce clinical malaria case incidence by 9.4% to 552 cases/1000 people/year, while administration to under 15 years old would reduce case incidence by 14.6% to 520 cases/1000 people/year.

## Discussion

Symptomatic malaria was rare in the cross-sectional survey and 91.9% of *P. falciparum* infections were asymptomatic which presents a challenge for malaria control since these individuals will not seek care and therefore infections will not be cleared^33^. We show a high burden of *P. falciparum* infection of 32.8% across all age groups at the end of the malaria transmission season in 2017 despite ITN universal coverage campaigns in 2010, 2013 and 2016 and malaria case management with ACTs. *P. falciparum* prevalence was 23.2% in children under 5 years old. *P. falciparum* prevalence was, however, highest in children aged 5 to < 10 years old (53.7%) and 10 to < 15 years old (56.7%) suggesting these ages are an important reservoir of infection in the community. While we used post-hoc age strata in this analysis, the large sample size was sufficient to identify significant differences between the under 5 years old, 5–10 years old and 10–15 years old children.

Administration of SMC to school age children aged 5 to 15 years could have a substantial impact on clinical malaria incidence in Burkina Faso. We show that against a background of pyrethroid-PBO ITNs, expanding the age group for SMC to 5–10 years old could avert an additional 57 cases/1000 people and to 10–15 years old could avert an additional 89 cases/1000 people/year in the Cascades Region of Burkina Faso. This is comparable to the additional 64 cases/1000 people/year predicted to be averted by switching from pyrethroid ITNs (673 cases/1000 people/year) to pyrethroid-PBO ITNs (609 cases/1000 people/year), assuming continued SMC administration to under 5 years old in the Cascades Region of Burkina Faso. The impact of expanding eligibility for SMC will depend on the type of ITN deployed in a region. Here we model the impact of SMC against a background of standard pyrethroid ITNs and pyrethroid-PBO ITNs. Further second generation ITNs are being rolled out, including for example, chlorfenapyr and alpha-cypermethrin ITNs. We lack field data to model the impact of chlorfenapyr and alpha-cypermethrin ITNs and so the models presented should be refined once the effectiveness of these ITNs is better understood.

Intermittent preventive treatment of malaria in school children (aged 5 to 15 years) has been trialled extensively across sub-Saharan Africa in both seasonal and perennial transmission settings with different treatment regimens (e.g. once, termly, monthly). A systematic review of intermittent preventive treatment of malaria in school age children found a 73% reduction in *P. falciparum* prevalence, 60% reduction in clinical malaria and 23% reduction in anaemia using study level meta-analysis of 13 studies conducted in West, Central and East Africa^34^. Individual participant data meta-analysis identified a marginal effect of intermittent preventive treatment in children aged 10–15 years on cognitive test scores, although no difference was found in all ages^34^. The review found intermittent preventive treatment to be well tolerated and acceptable to communities. School-aged children are significant reservoirs of human-to-mosquito transmission^35–37^. There is some evidence that administration of intermittent preventive treatment to school-age children can confer a community-level benefit to those not receiving intermittent preventive treatment. A clinical trial evaluating administration of SMC to children aged up to 10 years in Senegal showed a 26% reduction in malaria incidence in adults and children too old to receive SMC^38^. Our transmission dynamic models predict a 14.6% reduction in malaria incidence in all ages, which is probably a reflection of the higher transmission in our study setting compared to Senegal.

The potential benefit of expanding the age group eligible for SMC in Burkina Faso needs to be weighed against its potential risks, including development of drug resistance and/or the risk of hindering acquisition or maintenance of immunity. Cost implications and feasibility would also need to be considered. Use of SP-AQ for SMC in Burkina Faso avoids the first line treatment AL thus minimising potential harmful impacts should resistance develop. While theoretical models predict an ‘immunological deficit’ leading to higher burden in older children^39^, evidence from studies of intermittent preventive treatment in infants and children under 5 years old does not indicate this^40,41^. As malaria declines, the burden of malaria shifts to older children^28,42,43^ and so identification of interventions to protect these groups should be a priority. While expanding SMC and changing to pyrethroid-PBO ITNs will avert a considerable number of clinical cases, moving towards elimination in high transmission areas will require sustained coverage of existing interventions and development of additional interventions.

So far, Senegal is the only country in the Sahel that is administering SMC to children aged up to 10 years with clinical trials showing a high protective efficacy against malaria^38,44,45^. Delivery of SMC to children under 10 years old in Senegal is conducted by community health workers who conduct visits house-to-house. Interestingly, Bâ et al. found that while increasing the age group almost doubled the target population, it only increased the number of households to visit by 13%^45^. Whether community-based delivery would be feasible in Burkina Faso needs to be considered. School-based SMC delivery is another option^46,47^, although schools are often closed during the rainy season which can limit use of this delivery route during the peak transmission season^47^.

As well as age, this study identified several other important risk factors for malaria. Study participants who reported sleeping under an ITN the previous night had a 53% lower odds of *P. falciparum* infection after adjusting for all other risk factors. This suggests that bednets continue to provide personal protection in the study area despite high levels of insecticide resistance^13,48^. We also found evidence of an association between use of a fan and reduced *P. falciparum* infection, although sleeping in a room with an electric fan was not common in the study population (6.2%). As well as making sleeping under an ITN more comfortable, if sufficiently powerful fans will also discourage landing and feeding of malaria mosquitoes.

## Conclusion

*P. falciparum* infection in the Cascades Region of Banfora in Burkina Faso remains high despite universal coverage with ITNs and access to diagnosis and treatment. *P. falciparum* infection burden was concentrated in the 5–15 years old children and was predominantly asymptomatic. Additional interventions are needed to target this population group. Transmission dynamic modelling suggests that, even against a background of pyrethroid-PBO ITNs, administration of SMC to school age children may be able to substantially reduce malaria burden in south-west Burkina Faso and other countries in the region. Further research should be conducted to determine the feasibility, effectiveness and cost-effectiveness of SMC administered to 5–15 year old children in Burkina Faso and other countries in the Sahel.
